# Experiences of Patients With Auditory Processing Disorder in Getting Support in Health, Education, and Work Settings: Findings From an Online Survey

**DOI:** 10.3389/fneur.2021.607907

**Published:** 2021-02-18

**Authors:** Deepashri Agrawal, Giorgos Dritsakis, Merle Mahon, Alyson Mountjoy, Doris E. Bamiou

**Affiliations:** ^1^University College London Ear Institute, London, United Kingdom; ^2^Psychology and Language Sciences, University College London, London, United Kingdom; ^3^APD Support, London, United Kingdom; ^4^University College London Hospitals NHS Foundation Trust National Hospital for Neurology and Neurosurgery, Neuro-Otology, London, United Kingdom; ^5^National Institute of Health Research (NIHR) University College London Hospitals Biomedical Research Centre, London, United Kingdom; ^6^Audiological Medicine Department, Great Ormond Street Hospital, London, United Kingdom

**Keywords:** auditory processing disorder, patient survey, qualitative, patient experience, questionnaire

## Abstract

**Objective:** To explore the views and experiences of individuals with Auditory Processing Disorder (APD) and/or their families in getting support from services and to receive their suggestions for improvement.

**Design:** Cross-sectional random sample survey with descriptive analysis.

**Settings:** Online survey.

**Participants:** One hundred and fifty six individuals with APD and/or their family members from the APD Support UK patient support organization and four associated APD Facebook groups.

**Main Outcome Measure:** A 16-item questionnaire on negative and positive experiences in getting a referral for diagnosis, funding for the FM system, and overall support for APD.

**Results:** The key findings that emerged included reports of difficulty in getting a referral for diagnosis (54%), obtaining funding for an FM system (45%), getting support for APD (61%), and poor recognition and awareness of APD (63%) in Education, Health or Work settings. The positive experiences reported were ease in getting a referral for diagnosis (46%), in obtaining an FM system (20%), and with diagnosis leading to help at school or to a better understanding of the condition and the required adjustments. The recommended improvement areas were raising awareness of APD and related management in Education (30%), the Health sector (25%), and the public (18%).

**Conclusions:** Individuals and families of individuals with APD overwhelmingly report a lack of awareness of APD across health, education, and work sectors, and difficulties in getting access to diagnosis and support. This information may provide an initial understanding of the patients' needs for clinical services for APD, identify research priorities, and influence longer-term public health decisions toward improved care.

## Introduction

Auditory Processing Disorder (APD) is the commonly used term for the clinical presentation of listening difficulties in children and adults with normal audiograms but abnormal scores in complex psychoacoustic tests (1). It is classified under H93.25 in the current tenth version of the International Classification of Diseases (ICD-10). The subtypes according to risk factors include developmental APD, in children with a family history of language or developmental disorders and no other known risk factors (2) with symptoms that may persist into adulthood (3). Acquired APD may present after brain injury or stroke (4), or with aging (5, 6). Secondary APD may be present in individuals with a history of hearing impairment (2). Additionally, impaired perception of sound features corresponding to pathology in central auditory structures, that is disproportionate to the hearing levels, and not the direct result of a higher cognitive deficit, is a prominent feature of several genetic subtypes of dementia (7). APD may also be part of the phenotype in different schizophrenia subgroups (8) and may contribute to the psychiatric symptoms (9). Different aspects of non-speech sound processing show significant heritability (10, 11), and genetic work in the future may help to subclassify APD further in terms of the underlying pathophysiology.

The prevalence of APD was calculated to be 1.94 per 1,000 children by a recent retrospective study based on referrals and diagnoses made in a national audiology clinic (12). Hind et al. (13) estimated a prevalence of APD as 0.5–1% in the general population, based on a prevalence of normal audiometric findings. APD was prevalent in 5% of children and 0.9% of adults of all ages who were referred to a general audiology clinic (13). However, these estimates may be affected by ascertainment bias and the prevalence of APD is yet to be determined.

APD has a significant adverse impact on the affected individual's listening and communication, in both children (14) and adults (15). The condition often co-exists with developmental language (16), reading (17), attention disorders (18), in individuals with autism (19) and correlates with autism severity. Children with APD may differ from children with other developmental disorders but show similar performance on cognitive or language tests (20). In the UK education, these children are classified as having special educational needs (SEN). In 2018/19, only 30.4% of SEN children identified by year 11 achieved Level 2 by age 19, compared to 75.3% of non-SEN children (21). Children with APD and their parents report greater emotional difficulties, poor health, emotional and social skills than do normal-hearing children. The presence of a language disorder does not predict their psychosocial difficulties (22). Some children with APD have reported relying more on anger regulation and less on problem-solving as a coping strategy, but in the reported study, the sample size was small (*N* = 20) (23). Adults with APD similarly focus on restraining their emotions rather than on self-directed thinking (23, 24). APD in childhood may also affect the “sense of self” into early adulthood (3).

APD is diagnosed based on symptoms, poor performance on auditory processing tests, and consideration of other factors that may impact performance (25, 26). There are no uniform diagnostic criteria, with a varied diagnostic yield of criteria applied in previous studies (27). However, more recent consensuses and guidelines by several professional associations and societies appear to be better aligned (15, 28–32).

Management of APD may include listening exercises (i.e., auditory training) for children as well as adults (33, 34), metacognitive strategies (35), and remote microphone hearing aids (RMHAs). RMHAs may improve speech understanding by 53% (36). There is “moderate support” that RMHA systems use in the classroom improve children's speech perception and listening skills in that setting, with mixed evidence that they improve academic performance (37). There are similar reports of improved speech in noise perception with RMHAs in adults with neurological type APD (4, 38, 39).

Awareness about APD remains low. More than half of surveyed UK Audiologists report that they are poorly informed about APD (40), while 18% of South African Audiologists report receiving limited undergraduate training for APD, and 19% report difficulty accessing additional post-graduate training (41). Over 70% of ENT and 90% of GP UK professionals report limited awareness of APD and approximately half are unlikely to refer for further APD assessment (42). The majority of mainstream primary teachers similarly report “very poor” awareness regarding APD (43).

There are fewer than 10 dedicated APD clinics for children and only two for adults within the UK NHS. On the positive side, the UK Department for Education recommended standards for the acoustic design of schools classifies children with APD as having special hearing/communication needs. However, at present only half of the UK classrooms fully comply with these standards (44, 45). Anecdotal reports by parents of children with APD suggest that educational support provided to their children and teenagers is limited. Adult subjects with APD visiting the clinics similarly reported limited support in their workplace. To our knowledge, APD Support UK is currently the only UK organization providing support for those affected.

In this context, it is crucial to understand the challenges faced by patients and their families in accessing the diagnosis and management of APD and support after the diagnosis, to inform planning for education, research, and clinical services toward improved care in the long-term. The present study aimed to explore the views and experiences of individuals with Auditory Processing Disorder (APD) and/or their families about getting support from services and to receive their suggestions for improvement.

## Methods

### The Survey

Following a pilot study (see Appendix 1), a questionnaire with 16 questions (10 open-ended and 6 closed) about patient experience with APD was developed by the last author. The purpose of the questionnaire was to collect information about APD diagnosis, access to services, support, positive and negative experiences and suggestions for improvements. The questions were then reviewed by all authors, who are experts in the field of hearing impairments and patient-reported measures and pilot-tested by a parent of a child with APD for usability, relevance and ease of completion. Minor revisions were made (see Appendix 2; Table 1). The final version of the questionnaire was administered online using the web-based survey tool Opinio (Opinio 7.10, Copyright 1998–2019 ObjectPlanet). The survey was open from May to July 2019.

**Table 1:** Summary of questions asked in the questionnaire with respective question numbers.

Questions 1–6	Demographic information
Questions 7–10	Difficulties getting support from services
Question 11–12	Negative (11) and positive (12) experiences with professionals, family, and the public
Question 13–15	Recommendations for change and research
Question 16	Final comments

### Participants

Ethics approval was obtained by the UCL Research Ethics Committee (project ID 14813/001). Before completing the questions, participants provided informed consent. The online survey was circulated to a convenience sample through the APD Support UK Facebook groups (~2,300 members), for parents, teenagers, young adults, and adults. It was also posted on the APD Support UK website: https://apdsupportuk.yolasite.com/. Participation in the survey was anonymous and voluntary. Patients and/or relatives of patients with APD were invited to participate by following a web link to the survey.

### Analysis

Responses to questions 1–6 (demographic data) were analyzed using descriptive statistics and reported as n (%). Open-ended responses (questions 7–16) were analyzed qualitatively. Three a priori domains were used as per the questions of the survey: Difficulties, Positive Experiences, and Recommendations. Analysis then focused on identifying subdomains within each of these domains following the content of the questions with analysis methods and steps introduced by Braun and Clarke (46, 47). Domains and subdomains were initially generated by author DA, scrutinized with GD and finally reviewed by all authors and finalized after consensus. Analyses were completed using Microsoft Excel for Office 365 and NVivo Pro v12.

## Results

### Demographics (Questions 1–6)

One hundred and fifty six participants completed the survey. Of these, 20% had been diagnosed with APD themselves, whereas 78% completed the survey on behalf of their son or daughter. Two participants responded for their partners and one for their grandchild. As some participants reported that there was more than one person with APD in the family, the responses represented 165 APD patients in total (mean age: 18 years old, standard deviation: 12.9, age range: 6–65 years). The demographic information is provided in Table 2.

**Table 2:** Demographic data (responses to questions 1–6).

	**Total number of respondents (missing data)**	**Results (percentage** **of respondents)**
Age at time of survey (year of birth)	165 (0)	Adults (28%); Children (72%)
Age at diagnosis	165 (0)	6–17 years (74%); 18–30 years (12%); 31–40 years (4%); 41–50 years (6%); 51–65 years (4%)
Diagnosis of APD	152 (4)	Made by Audiologist (50%), Neuro-otologist/Neuro Audiologist (13%), Speech and Language Therapist (10%), Ear Nose Throat specialist (8%), Psychologist (6%), Pediatrician (4%), Neurologist (3%), Education specialist (3%), Teacher of the deaf (2%).
Setting of diagnosis	156 (0)	Hospital (74%), Private clinic (15%) School (4%), College (1%), University clinic (6%).
Additional diagnosis	156 (0)	None (9%); Dyslexia (22%); Autism (18%); Sensory Processing Disorder (14%); Visual Processing Disorder (12%); Hearing loss (10%); ADHD (8%); Other [Tinnitus (3%); hyperacusis (2%); Dyspraxia (1%)]

### Qualitative Data Analysis

**Figure 1:**
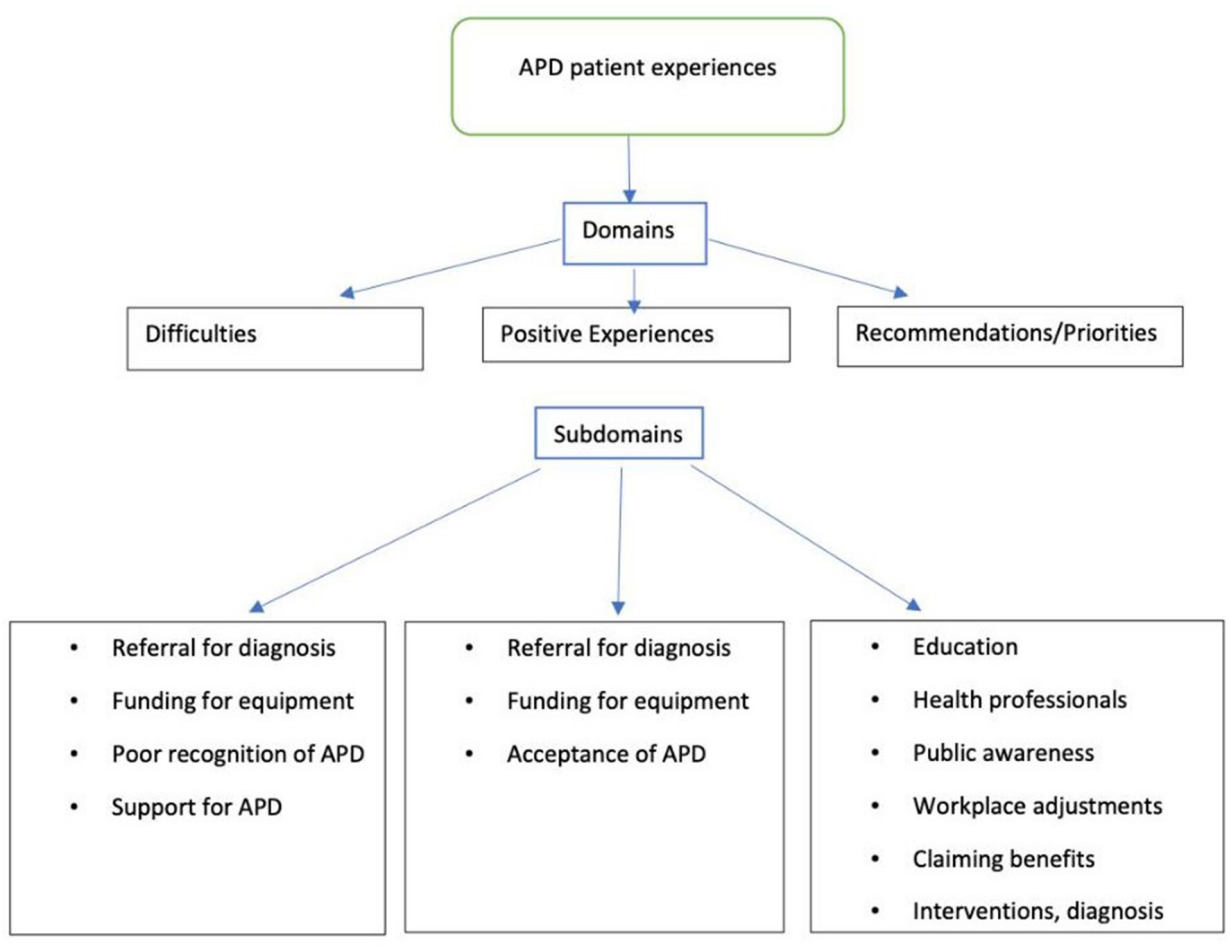
Subdomains identified within each of the 3 a priori domains, illustrating the experiences of people diagnosed with APD, and their families who participated in the present study.

The results of the descriptive analysis of the responses to questions 7–16 can be seen in Figure 1. As explained above, subdomains were identified within the 3 a priori domains. In the “Difficulties” domain, the subdomains corresponded to the survey questions (7–11), while for the other 2 domains (Positive Experiences and Recommendations/priorities) subdomains focused on questions 12 and questions 13–15, respectively. The responses are reported as percentages when relevant and informative. Domains and subdomains are presented below alongside illustrative quotes. Responses to specific domains and subdomains have been quoted in Tables 3, 4.

**Table 3:** Responses to the Domain: Difficulties faced, with the percentage of responses for each subdomain, and illustrative statements given by the respondents.

**Questions/subdomains**	**Difficulties: Yes (%)**	**Difficulties reported**	**Illustrative statements**
7 Diagnosis Referral	84 (54%)	Convincing GP about problem 50%	“GPs kept dismissing me and my son. This went on for 5 years.” (Mum of 7 years old boy with APD)
8 Equipment funding	70 (45%) [N/A 56 (35%)]	APD not recognized; Schools reluctant to consider RMHAs	“School wouldn't even apply due to funding. Couldn't get funding from deaf society as not hearing impaired” (Mum of 8 years old girl with APD).
9 APD Recognition	99 (63%)	Education 55% (awareness/recognition/reluctance for adjustments) Work 9% (no or limited adjustments) Health 11% (low awareness or skepticism) Claiming Benefits 7%	“Enormous difficulty in getting her (sic) child's mainstream Secondary School to recognize/accept auditory processing difficulties. They have no understanding of APD at all.” (Mum of 7 years old boy with APD) “Needs not recognized at work. Took a period of 4 months sick leave for my employer to recognize that something needed to change.” (40 years old individual with APD) “Medical professionals such as Pediatrician and ENT Consultants very skeptical about possible APD relevance and validity as a diagnosis.” (Mum of 8 years old boy with APD) “I applied for Disability benefit/and was turned down” (41 years old Individual with APD)
10 APD recognition after diagnosis	96 (61%) (8 missing data)	Health 39% Education 27% Work 33% Other 1%	“Yes, he keeps being told to listen, stop daydreaming, stop asking for help, get on with your work or you will be kept in. APD is that made up never heard of it.” (Mum of 9 years old boy with APD). “Family/friends don't understand why we turn down some invitations that we know will cause stress e.g., noisy party environments. Strangers have commented about her behavior.” (Mum of 8 years old girl with APD). “With anyone, even my own family and partner they either don't believe it and think i am being ignorant or forget.” (Female, 26 years old with APD)
11 Negative experiences	156 (100%)	Health/other professionals 59% Family 24% Strangers 11% Work 3% Benefit advisers 3%	“Teacher didn't understand APD, end of year reports always put daughter down for APD. Teacher sat her facing the wall so she did not get distracted, I complained” (Parent of 11 years old girl with APD) “Both friends and family did not like me asking them constantly to repeat themselves” (18 years old individual with APD) Strangers “People think her response is rude, she just hasn't heard!” (Mother of 13 years old with APD) “Colleagues have been cruel at times, mocking. I have challenged this where I could.” Benefit Advisers “Student Finance England received my diagnosis letter for APD and said it was not enough evidence of disability for me to receive DSA.” (20 years old individual with APD)

**Table 4:** Responses to the Domain: positive experiences, with percentage for each subdomain and illustrative statements given by the respondents.

**Subdomains**	**Percentage**	**Reported experiences**	**Illustrative statements**
Referral for diagnosis	46%	Through school 36% By going privately 36% Through GP 24%	“Gosh, doctors were great. Some teachers and the SENCO were great.” (Parent of 8 years old girl with APD). “Our GP knew about APD.” (Father of 8 years old boy with APD)
Funding for equipment/support at school	20%	Local authority helped/system borrowed 40% Funded by research 20% School made adjustments 40%	“School insisted on APD interventions in EHCP No problem getting DLA for my son (he has other comorbidities)” (Mum, of 9 years old boy with APD).
Acceptance of APD	34%	Family/friends understand APD 25% Work considerate of APD 5% Teachers/SENCO/Tutors 30% Audiologist 5% University Doctors and GPs 35%	“We as a family understand the need to provide xxx with a calm, quiet learning environment and try our best to allow him as much time as needs to process information.” (Parent of 10 years old boy with APD) As a Managing Director of my own company, I work with Headteachers. The staff team and Headteacher totally gets my APD. I am open about it feel valued and accepted” (40 years old female with APD) “Teachers have always made a huge effort to understand APD and have been creative in finding ways to support my daughter, particularly in relation to self confidence and esteem.” (Parent of 9 years old girl with APD) “Audiologist was very informed and supportive” (Parent of 11 years old boy with APD) “Gosh doctors were great.” (Parent of 8 years old girl with APD) “My own GP was fantastic when we were first trying to get a diagnosis. Very supporting and even though he stated he didn, '´Aôt have a lot of experience or knowledge about APD he went away and did loads of research and has guided and supported us throughout our journey.” (Parent of 9 years old boy with APD)

#### Domain: Difficulties (Questions 7–11)

Respondents reported difficulties in getting a referral (54%), in getting funding for the recommended equipment (24%), whereas (63%) reported difficulties in getting APD recognized and accepted, and difficulties getting support for APD. All respondents reported negative experiences in interacting with others (Table 3).

#### Domain: Positive Experiences (Question 12)

Positive experiences were identified by 150 respondents in terms of subdomains of referral for diagnosis (46%), funding for equipment/support at school (24%), and acceptance of APD (36%) (Table 4).

#### Domain: Recommendations for Improvement (Questions 13–15)

Questions 13–15 (156 respondents) had overlapping responses and were divided into different subdomains as presented below.

##### Subdomain: Educating/Training

Thirty percentage of respondents suggested prioritizing the education of teachers regarding APD including coping strategies for APD, use of the equipment, and development of screening tests for school-aged children.

“*More understanding by teaching professionals at a local level*> *NHS SALT looking at the big picture rather than just identifying speech sound difficulties”*

*(Grandmother of a 6 years old boy with APD)*“*Make it as important as children with dyslexia and autism”*

(Parent of 9 years old child with APD)

##### Subdomain: Training Health Professionals

Twenty five percentage of recommended training for medical professionals regarding APD and the importance of RMHAs.

“*Hearing aids, FM systems should be funded in schools”*

(Parent of 11 years old girl with APD)

##### Subdomain: Public Awareness

Eighteen percentage of suggested raising public awareness.

“*More public awareness! More cross-agency working”*

(Mum of 11 years old boy with APD)

##### Subdomain: Workplace Adjustments

Six percent suggested educating employers.

“*Rather than good practice, it needs to be an essential practice that any employer is made aware of that person's disability and that reasonable accommodations are made to put them on an equal footing with their colleagues”*

(40 years old individual with APD)

##### Subdomain: Claiming Benefits

Seven percent recommended focusing on claiming benefits.

“*CLAIMING BENEFITS 1. Qualified DWP government assessors to assess, NOT for-profit private enterprise assessors 2. Advocates for people with APD 3. Working knowledge of APD so that assessors don't make assumptions about what people with APD can and cannot hear etc. 4. Filming of assessments so that unscrupulous assessors cannot take advantage of people with APD.”(Parent of 13 years old boy with APD)*

##### Subdomain: Research Into Interventions, Diagnosis, and Comorbidities

Out of 136 respondents, 59% recommended research focused on management, 31% on APD diagnosis, and 29% on symptoms and comorbidities.

“*Effectiveness of Interventions—clinical evidence—research into auditory training programmes, computer-based learning programmes such as Earobics (recommended but not commercially available) or Fastforword, etc.”*

*(Parent of a 7 years old child with APD)*.

“*Early diagnostic processes in schools. More trained audiologists to diagnose locally. Research as to better diagnosis and coping strategies”*

(Parent of 12 years old with APD)

“*Research on other combined issues that go hand in hand with APD”*

(Parent of 7 years old with APD)

Thus, respondents reported, difficulty in getting a referral for diagnosis (54%), obtaining funding for an FM system (45%), getting support for APD (61%). The positive experiences reported were ease in getting a referral for diagnosis (46%), in obtaining an FM system (20%). The recommended improvement areas were raising awareness of APD and related management in Education (30%), the Health sector (25%), and the public (18%).

## Discussion

Poor awareness and understanding of APD were reported by the majority of respondents of the present survey, particularly in professionals in relevant positions, in line with previous reports by surveyed professionals (41–43). This lack of knowledge among medical professionals is of concern, as it may lead to delayed diagnosis and management of APD in affected individuals, and failure to meet the guidelines of the National Service Framework (NSF) for “early recognition, prompt diagnosis, and treatment” (48). Children faced difficulties at school due to poor awareness/recognition of APD, despite the classification of APD as requiring SEN support by the Department for Education (44). Similarly, participants reported poor recognition and no support for APD at work. Reasonable adjustments both in education and in the workplace are a legal requirement.

The majority of respondents had negative experiences in getting RMHAs, with funding and other obstacles, despite the NSF requirement for people with long term conditions to receive “timely, appropriate assistive technology/equipment,” (48). RMHAs improve listening and attention skills in children (8), and adults with neurological-type APD (4, 38, 39), and are thus a reasonable adjustment as per the Equality Act 2010 (49). Our results highlight the need for teachers, in particular, to be better informed about APD (41, 43) not least because they may be the first to notice gaps in a child's language, social behavior, and communication skills (25).

The positive experiences reported by the participants were mixed, with some reporting that they did not have difficulty getting a diagnosis and with the diagnosis leading to help or a better understanding of their condition. The three key prioritized improvement areas were raising awareness of APD in Education and Health, reflecting concerns by Audiologists that they have access to limited training on APD (41). The identified research priorities were focused on intervention, diagnosis, and comorbidities of APD. These are in line with studies showing that APD overlaps with developmental disorders (20), reports that few audiologists treat APD (50), and that there are just a handful of studies on intervention benefits (51).

Results of the present patient survey indicate that there is little that has changed for patients and families with APD in the past 10 years. There is a need for continued discussion toward a definition of APD [e.g., (14)] that will be universally accepted. On the positive side, a definition of APD was discussed during the forthcoming ICD 11 beta version development, with the APD entry (under AB5Y Other specified disorders with hearing impairment) accepted by the International Federation of ORL Societies (52). There is a need for training not only for Audiologists, whose curricula already include APD, but also for teachers and general practitioners. Encouragingly, some information on APD is included on the NHS site (53), but further formal training is required. Guidelines and pathways for the provision of remote microphone hearing aids need to be agreed upon by the Health and Education sectors and established.

The study has some inherent limitations, notably that the authors had no quality control on the diagnosis of the individuals taking part in the survey. The convenience sample from four Facebook groups may not be representative of the broad UK population. There was no follow up with a face-to-face interview to investigate the domains and subdomains identified in more detail or depth. We did not look at socioeconomic factors or audiological data, to maintain patient anonymity. The potential bias in participant responses, which is an inherent limitation of qualitative research as a whole, also has to be mentioned. Nevertheless, this is the first UK survey of subjects with APD and their families and is consistent with previous surveys of professionals in Health and Education. These subjective reports have real-life validity and could contribute to planning for NHS services by giving people choice, through services planned and delivered around their individual needs, inform clinical decision making and guide future research.

## Conclusions

In conclusion, individuals with APD and their families overwhelmingly reported difficulties in getting access to diagnosis and support. They request increased awareness of APD across health, education, and work sectors. Despite the study's limitations, this information may provide some initial understanding of the needs of the individuals with APD and their families. There is a pressing need to improve awareness and recognition of APD and address what patients perceive as research priorities.

## Data Availability Statement

The raw data supporting the conclusions of this article will be made available by the authors, without undue reservation.

## Ethics Statement

The studies involving human participants were reviewed and approved by UCL Research Ethics Committee (project ID 14813/001). The patients/participants provided their written informed consent to participate in this study.

## Author Contributions

DA performed the data analysis, literature review, interpretation of results, drafted the first version of the manuscript, and is responsible for submitting the manuscript. GD contributed toward developing the questionnaire, study design, data collection, data analysis, and editing the manuscript. AM is a patient collaborator who contributed equally with the other authors in research design, contact, analysis, and interpretation/write up. MM contributed to the editing of the manuscript. DB is responsible for designing the study, study coordination, data analysis and interpretation, and the editing and final version of the manuscript. All authors have contributed equally and approved the final manuscript.

## Conflict of Interest

The authors declare that the research was conducted in the absence of any commercial or financial relationships that could be construed as a potential conflict of interest.
