# Supplementary material for: Experiences of Patients With Auditory Processing Disorder in Getting Support in Health, Education, and Work Settings: Findings From an Online Survey
Source: Front Neurol. 2021 Feb 18;12:607907. doi: 10.3389/fneur.2021.607907 (PMC7930331; doi:10.3389/fneur.2021.607907)
Supplement: Supplementary file 1 [file Data_Sheet_1.PDF]

Appendix 1 – Pilot

In 2013, a 9-item open-ended questionnaire about experiences with APD (developed by the last author) was administered to families at the APD awareness day hosted at the National Hospital of Neurology and Neurosurgery (NHNN) and Great Ormond Street Hospital (GOSH) in London, UK, that was attended by 32 adults (APD patients and/or parents or family members). The audience was split into 6 groups (one group of 4 and six groups of 5 patients or family members). Participants in each group were given 15 minutes to fill in the questionnaire and 15 minutes to discuss the questionnaire results within the group. Written responses from 9 adults or relatives of adults with APD and 17 parents or relatives of children with APD were collected in these small groups and then presented by each group leader to the audience. Respondents identified the following key issues (collated from the questionnaires and subsequent discussion minutes): late and long/difficult journey to diagnosis; lack of awareness of APD; lack of related support (). Positive experiences reported included help provided after diagnosis predominantly within the education setting, a "better understanding of the problem" and adjustments/acceptance.

1. My own /My family's APD story (please write 2-3 sentences, you will have 3 min to introduce yourself to your group)

2. Negative experiences (please list up to 2-3 under each heading in order of importance)
    a. With health professionals/education professionals

    b. With public including family and friends

3. Positive experiences (please list up to 3 in order of importance)

4. What I would like to change (please list up to 3 in order of importance)

5. Priority areas in research (please list up to 3 in order of importance)

6. Priority areas in public policy(please list up to 3 in order of importance)

7. Any other comments?

Appendix 2 – The survey questionnaire

Questions are categorised into the 4 broad domains that were used for analysis and reporting of the results.

Demographic information	1. Who is diagnosed with APD in your family?<br>a. Yourself<br>b. Your son/daughter, husband/wife or other family members (please say which one)<br><br>2. Year of birth of a person with APD<br><br>3. Age at diagnosis of APD<br><br>4. Who made the diagnosis:<br>a. Audiologist<br>b. Neurologist<br>c. Paediatrician<br>d. Speech and Language Therapist<br>e. Educational Psychologist<br>f. ENT<br>g. Other - please specify<br><br>5. Where was the diagnosis made?<br>a. Hospital<br>b. GP<br>c. School<br>d. Other – please specify

	6. Do you / your family member with APD have any other diagnosis of additional disabilities or conditions or medical or other needs? Please tick all that apply:<br>a. None<br>b. Autism<br>c. Downs Syndrome<br>d. ADHD<br>e. Language Disorder<br>f. Visual Processing Disorder<br>g. Sensory Processing Disorder<br>h. Dyslexia<br>i. Dyspraxia<br>j. Hearing Loss<br>k. Other – please specify
Difficulties in getting services and support	7. Did you/your family member with APD have any difficulties in getting a referral for APD testing?<br>a. No<br>b. Yes – please say what these difficulties were<br><br>8. Did you/your family member with APD have any difficulties in getting funding for recommended equipment following diagnosis (e.g. FM system)?<br>a. N/A<br>b. No<br>c. Yes – please say what these difficulties were below<br><br>9. Did you/your family member with APD have any difficulties in getting recognition/acceptance of APD by professionals following diagnosis, whether in the health setting, in education or at work or when seeking work/claiming benefits?

	a. No<br>b. Yes - please say what these difficulties were<br><br>10. After diagnosis, did you/your family member with APD have any difficulties in getting support for APD in the health setting; education; at work; other?<br>a. No<br>b. Yes – please say what these difficulties were<br><br>11. Have you /your family member with APD, had any other negative experiences related to APD?<br>Please give the experiences that were most important for you in the box below<br>a. With health professionals/education professionals<br>b. With family and/or friends (please state which)<br>c. With strangers/in public<br>d. With employers/work colleagues (please state which)<br>e. With benefits advisers or other professionals (please state which).
Positive experience in getting services	12. Have you / your family member with APD had any positive experiences related to APD? Please explain below.<br>a. With health professionals/education professionals<br>b. With family and/or friends (please state which)<br>c. With strangers/in public<br>d. With employers/work colleagues (please state which)<br>e. With benefits advisers or other professionals (please state which).
Suggestions for improvement	13. What do you think are the most important areas for research in APD? Give 2 or 3 suggestions that are most important for you starting from the most important and working down the list.

	14. What would you like to change to better support people with APD? For each heading, give up to 2 or 3 suggestions that are most important for you<br>a. In health<br>b. In education<br>c. In public awareness<br>d. In the workplace<br>e. In claiming benefits<br>f. With other professionals (please state which).<br><br>15. What do you think are the most important areas for public policy relating to APD?<br>For each heading, give up to 2 or 3 suggestions that are most important for you:<br>a. In health<br>b. In education<br>c. In public awareness<br>d. In the workplace<br>e. In claiming benefits<br>f. With other professionals (please state which).<br><br>16. Any other comments?
